# Accuracy and reliability of accelerometer-based pedometers in step counts during walking, running, and stair climbing in different locations of attachment

**DOI:** 10.1038/s41598-024-78684-w

**Published:** 2024-11-12

**Authors:** Jiahao Pan, Shutao Wei

**Affiliations:** 1 Biomedical Engineering Doctoral Program, Boise State University, Boise, 83706 USA; 2 Department of Physical Education, Xiamen University of Technology, Xiamen, 361000 China; 3 361° (CHINA) CO., LTD, Xiamen, 361009 China

**Keywords:** Physical activity, Exercise medicine, Exercise track, Step-counting, Wearing positions, Disease prevention, Health services, Quality of life

## Abstract

This study aimed to investigate the accuracy and reliability of accelerometer-based pedometers placed on the wrist, waist, and shoe’s midsole during walking, running, and stair climbing. Twenty healthy adults were recruited. Steps were recorded by the pedometers and visually assessed from simultaneously recorded video to evaluate the accuracy of each pedometer in different locations of attachment. One week later, steps were recorded again by the pedometers only to evaluate the reliability of each pedometer in different locations of attachment. The wrist-worn pedometer presented significantly greater error scores compared to the midsole-worn pedometer during walking (*p* < 0.001), running (*p* = 0.006), and stair climbing (*p* = 0.003). Additionally, mean absolute precent error and Bland-Altman plots indicated that the pedometer worn in the midsole was most accurate for running and stair climbing, followed by waist-worn and wrist-worn pedometers. Furthermore, the midsole-worn and waist-worn pedometers showed strong reliability during walking and running, but only the midsole-worn pedometer presented acceptable reliability during stair climbing. The pedometer’s position impacts the accuracy and reliability of step counts, especially for walking and stair climbing. Embedding the pedometer into the midsole seems an effective approach to improve the accuracy and reliability of step counts.

**Supplementary Information:**

The online version contains supplementary material available at 10.1038/s41598-024-78684-w.

## Introduction

Regular physical activity is associated with improved health and well-being in human populations^1,2^, contributing to the prevention of metabolic diseases^3,4^, cardiovascular diseases^5^, infectious diseases^6^ and so on. Walking 10,000 steps/day has become a commonly-acknowledged goal for daily physical activity in adults^7–9^. Wearable devices, including pedometers, have been widely used to help individuals meet or exceed the recommended daily physical activity^10–13^. For example, it has been demonstrated that using pedometers can increase individuals’ daily activity by more than 2,000 steps/day when aiming for the 10,000 steps/day goal^11^. Furthermore, pedometers can be utilized to monitor national and international trends in physical activity^14^. The data obtained from physical activity monitors can be applied to intervention programs aimed at promoting well-being and preventing and treating diseases. Therefore, pedometers are highly recommended for commercial and research purposes.

There is a wide range of commercial pedometers typically worn on the wrist and waist to estimate steps. The advantages of wrist-worn pedometers include longer wear times compared to waist-worn pedometers, as they are more comfortable to wear and individuals are less likely to remove them during the day and night^15,16^. In addition, a national cross-sectional survey indicated that the wrist was considered the most popular location for attaching pedometers, with accuracy the most important characteristic^17^. However, the wrist-worn pedometers generally present lower accuracy in estimating steps that is evaluated by the mean precent error, mean absolute precent error, and Bland-Altman plots^16,18,19^. For instance, one study indicated that the wrist-worn pedometers underestimated the number of steps by 2.7–10.2% during walking^18^. Additionally, a review article summarized that the most accurate wrist-worn pedometer in estimating steps across 72 devices from 29 brands is the Fitbit Charge (Fitbit Charge HR), but it still presents a mean absolute percent error of about 25%^19^. Compared to the wrist-worn pedometers, the waist-worn pedometers have higher accuracy in estimating steps during activities^18,20–23^. For example, a study observed that the waist-worn pedometers underestimated steps by only 0.4% for a five-minute walk^18^. However, study also reported that the waist-worn pedometers had low accuracy in estimating steps, which indicated a mean absolute percent error of 6.9 to 11.1% in step counting for ten minutes of walking and running^20^.

On the other hand, pedometers attached on body positions above ankle joint are not recommended for estimating steps during stair climbing due to their low accuracy^24–26^. For example, prior work indicated that the mean error in estimating steps during ascending & descending 500 stairs is 10.01% for a pedometer placed on the anterior aspect of mid-thigh, 7.88% for a pedometer placed on the anterolateral aspect of middle shank, and 10.70% for a pedometer placed on the lateral aspect of distal shank^25^. In addition, the mean absolute precent errors of waist-worn pedometers in estimating steps during stair ascent and stair descent are 19.9% and 10.8% respectively^24^. Stair climbing mainly produce the altered kinematic characteristic of foot during the swing phase^27^, which may present higher sensation to detect the change of foot acceleration when using the accelerometer attached to the shoe compared to other body positions. Therefore, it is promising to enhance the accuracy of step counts embedding pedometers into the shoe midsole. Furthermore, embedding pedometers into the shoe’s midsole not only improves comfort but is also easier to use compared to wrist- and waist-worn pedometers^28^. However, there is insufficient experimental evidence to declare the accuracy and reliability of midsole-worn pedometers in estimating steps during physical activity^29^.

The purpose of this study was to determine the accuracy and reliability of pedometers in estimating steps when placed in different locations of attachment (wrist, waist, and midsole) during walking, running, and stair climbing. We hypothesized that (1) higher accuracy and reliability would be observed in the waist- and midsole-worn pedometers compared to wrist-worn pedometer during walking and running; and (2) higher accuracy and reliability would be observed in the midsole-worn pedometers compared to wrist- and waist-worn pedometers during stair climbing.

## Results

**Fig. 1:**
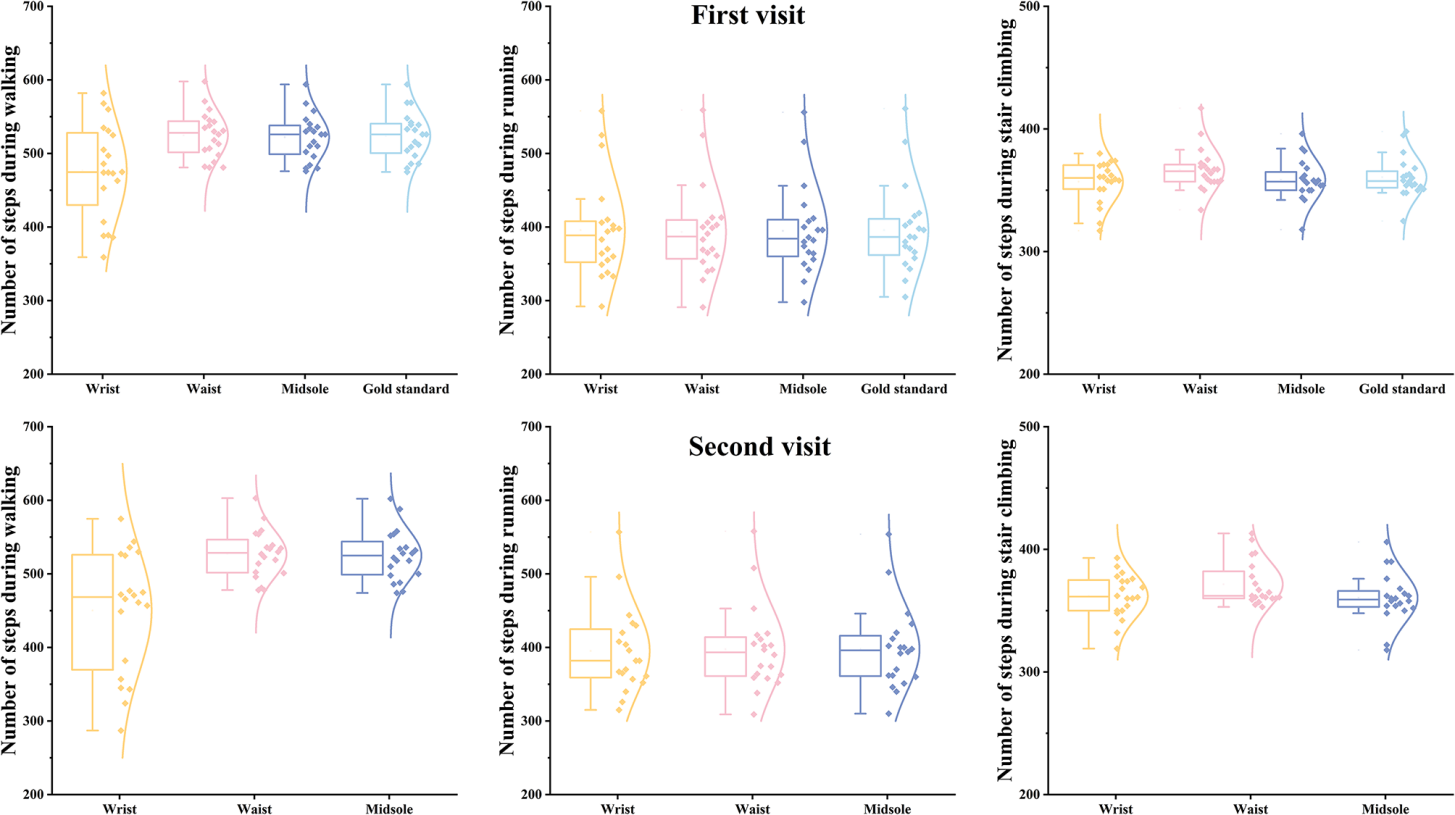
Number of steps measured by pedometers at different locations of attachment (wrist, waist, and midsole) and by the video camera (gold standard) during walking, running, and stair climbing at first and second (no gold standard recorded) visits.

In the current study, 20 healthy young adults (10 male/10 female; age: 27.0 ± 4.1year; height: 167.3 ± 6.6 cm; weight: 58.4 ± 9.4 kg; body mass index: 20.8 ± 2.4 kg/m^2^) participated. The average walking and running speeds were 1.51 ± 0.12 m/s and 2.90 ± 0.40 m/s in the first visit, respectively. Figure 1 illustrated the median (IQR) number of steps as recorded by the three pedometers and the gold standard at three different locations of attachment (wrist, waist, and midsole) during walking, running, and stair climbing from the first and second (without the gold standard) visits.

**Fig. 2:**
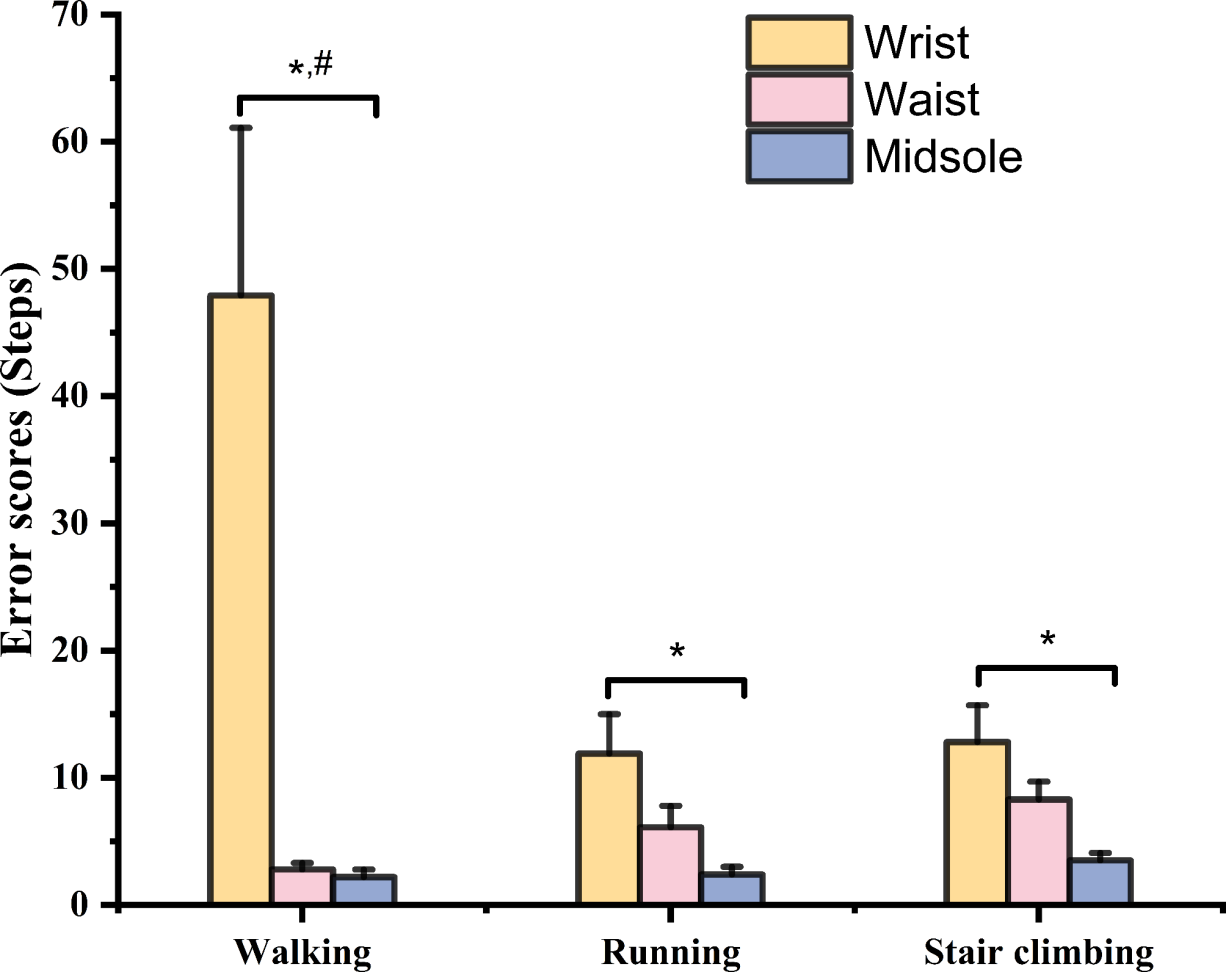
The error scores (mean ± standard error) at different locations of attachment (wrist, waist, and midsole) during walking, running, and stair climbing. “#” represents significant difference between wrist- and waist-worn pedometers; “*” represents significant difference between wrist- and midsole-worn pedometers.

Figure 2 presented difference in error scores at different locations of attachment (wrist, waist, and midsole) during walking, running, and stair climbing. Significant differences in error scores were observed during walking (F_2, 57_ = 11.789, *p* < 0.001), running (F_2, 57_ = 5.399, *p* = 0.007), and stair climbing (F_2, 57_ = 6.106, *p* = 0.004). Post hoc tests indicated that the wrist-worn pedometer had significantly greater error scores than both the waist- (*p* < 0.001 & Cohen’s d = 1.08) and midsole-worn (*p* < 0.001 & Cohen’s d = 1.09) pedometers during walking. Additionally, the wrist-worn pedometer presented greater error scores than the midsole-worn pedometer during running (*p* = 0.006 & Cohen’s d = 0.93) and stair climbing (*p* = 0.003 & Cohen’s d = 0.99). There was no significant difference in error scores between the waist-worn and midsole-worn pedometers during walking, running, and stair climbing (*p* > 0.05).

Table 1 displayed mean precent error, mean absolute percent error, and 95% confidence interval from the wrist-, waist-, and midsole-worn pedometers during walking, running, and stair climbing. The wrist-worn pedometer presented greatly underestimated the number of steps, with midsole-worn underestimating albeit to a much lesser extent, followed by waist-worn during walking. During running, our observations made it difficult to identify the overestimation or underestimation of the number of steps during running since the 95% confidence interval includes zero. The midsole-worn pedometer slightly underestimated the number of steps, whereas the waist-worn pedometer exhibited a slight overestimation of the number of steps during stair climbing. Furthermore, the wrist-worn pedometer presented greater total error in estimating the number of steps compared to waist- and midsole-worn pedometers during walking. Additionally, the pedometer worn in the midsole was the smallest total error in estimating the number of steps, followed by waist-worn and wrist-worn pedometers during running and stair climbing.

**Table 1:** Mean percent error (MPE, %), mean absolute precent error (MAPE, %), and 95% confidence interval (95% CI) from the wrist-, waist-, and midsole-worn pedometers during walking, running, and stair climbing.

	Walking			Running			Stair climbing		
	MPE	MAPE	95% CI	MPE	MAPE	95% CI	MPE	MAPE	95% CI
Wrist	− 9.04	9.09	(− 14.26, − 3.82)	− 0.21	2.98	(− 2.30, 1.87)	− 0.91	3.50	(− 3.19, 1.38)
Waist	0.18	0.53	(− 0.13, 0.48)	− 0.71	1.60	(− 1.91, 0.49)	1.70	2.30	(0.61, 2.78)
Midsole	− 0.37	0.42	(− 0.61, − 0.14)	− 0.32	0.61	(− 0.75, 0.11)	− 0.55	0.98	(− 1.08, − 0.027)

**Fig. 3:**
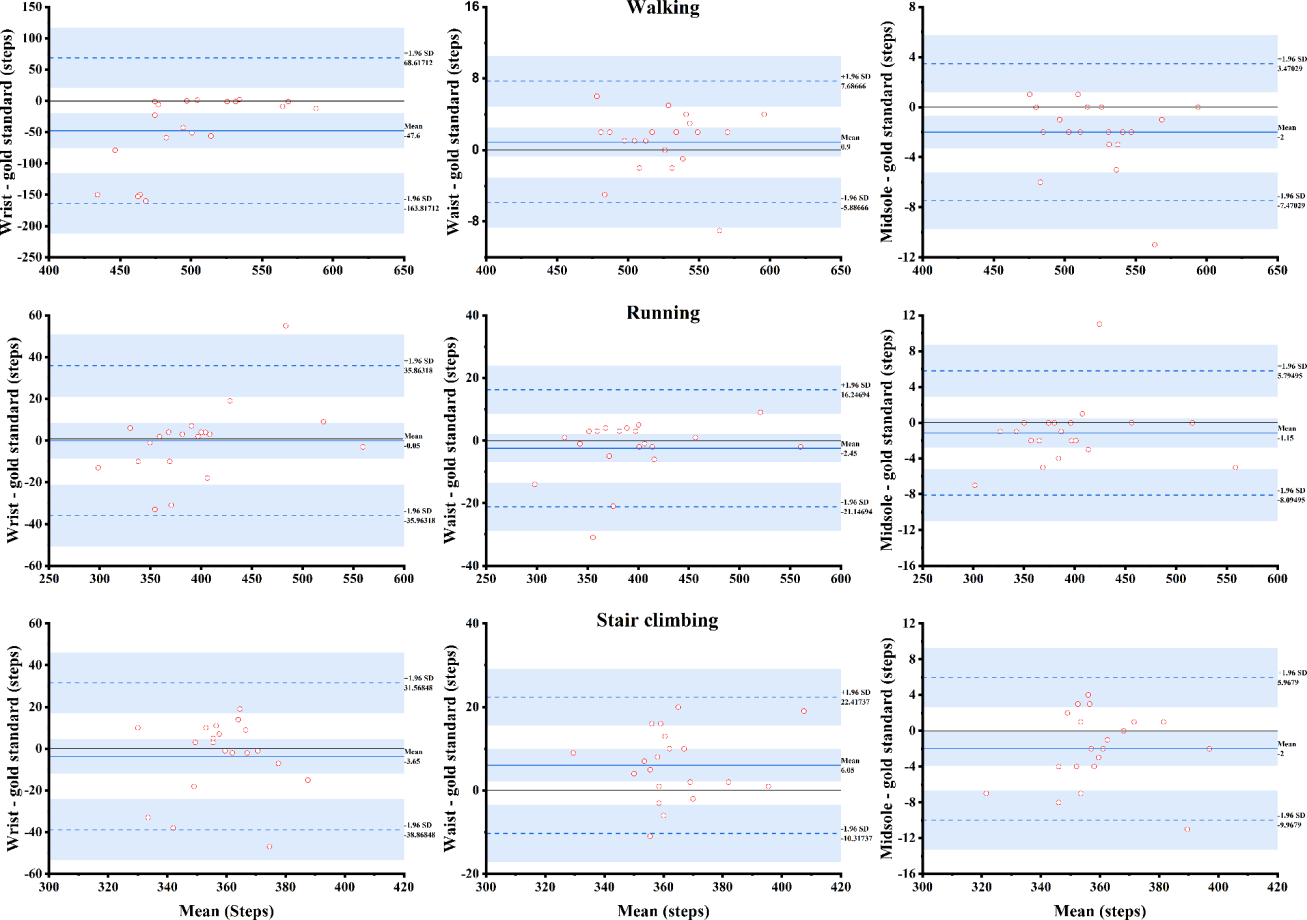
Bland-Altman plots comparing the number of steps form the pedometers at different locations of attachment (wrist, waist, and midsole) and the video camera (gold standard) during walking, running, and stair climbing. Solid horizontal black line represents zero; blue dash line represents 95% prediction intervals.

Figure 3 illustrated the level of agreement between the pedometers at different locations of attachment and the gold standard during walking, running, and stair climbing. The midsole-worn pedometer presented remarkable accuracy during each condition, with the 95% prediction intervals within ± 10 steps from zero. The waist worn pedometer presented similar accuracy to the midsole-worn pedometer during walking, with the 95% prediction intervals within ± 10 steps from zero. However, the accuracy of waist-worn pedometer decreased during running and stair climbing, with the 95% prediction intervals within ± 25 steps from zero. Additionally, the waist-worn pedometer tended to slightly underestimate the number of steps during running, whereas presented slight overestimated the number of steps during stair climbing. The wrist-worn pedometer showed lower accuracy compared to the waist- and midsole-worn pedometers in all conditions, especially during walking.

Table 2 showed the intra-device reliability values from the wrist-, waist-, and midsole-worn pedometers during walking, running, and stair climbing. The results reported that the waist- and midsole-worn pedometers had strong reliability during walking and running. The wrist-worn pedometer presented strong reliability during running, but it only showed acceptable reliability during walking. Additionally, only the midsole-worn pedometer showed acceptable reliability during stair climbing.

**Table 2:** Cronbach’s alpha value from the wrist-, waist-, and midsole-worn pedometers during walking, running, and stair climbing.

	Walking	Running	Stair climbing
Wrist	0.82	0.95	0.40
Waist	0.93	0.97	0.65
Midsole	0.93	0.98	0.79

## Discussion

The aim of this study was to investigate the accuracy and reliability of pedometers placed at different positions (wrist, waist, and midsole) to measure steps during walking, running, and stair climbing. First, our study observed that the accuracy of the pedometers followed the order of the midsole-worn > waist-worn > wrist-worn during running and stair climbing. In addition, the wrist-worn pedometer presented significantly greater error scores and underestimated the number of steps compared to the waist- and midsole-worn pedometers during walking. Furthermore, both the midsole-worn and waist-worn pedometers showed strong reliability during walking and running, but only the midsole-worn pedometer presented acceptable reliability during stair climbing. The wrist-worn pedometer presented strong reliability during running and acceptable reliability during walking. These observations were consistent with our hypothesis.

Many previous studies have demonstrated that the waist-worn pedometers have higher accuracy and underestimated the number of steps compared to the wrist-worn pedometer during walking^18,22,23^, which aligns with our observations. For instance, one study reported that significantly fewer steps were recorded by the wrist-worn compared to waist-worn pedometers (5,877 vs. 6243 steps) during 30 min of treadmill walking^22^. Another study observed that the error scores of two wrist-worn pedometers (55.00 ± 42.58 & 43.50 ± 49.71 steps) were greater than two waist-worn pedometers (28.58 ± 33.86 & −3.83 ± 22.05 steps) when walking at a treadmill for 5 min at a speed of 3.5 mph^20^. Additionally, wrist-worn pedometers underestimated the number of steps by 2.7 to 10.2%, whereas the waist-worn pedometers presented only a -0.4% underestimation during 5 min of walking^18^. This possible explanation is that some participants may not walk quickly with minimal arm movements, leading to decreased accuracy in step counting during walking^30^. Similar to the waist-worn pedometer, the midsole-worn pedometer also presented higher accuracy in counting steps compared to the wrist-worn pedometer during walking. Although no direct study comparison the accuracy in estimating steps between wrist-worn and midsole-worn pedometers, prior works indicated that the ankle-worn pedometer presents higher accuracy and reliability compared to the wrist-worn pedometers during walking^25,31,32^. In the current study, therefore, it is reasonable to observe the strong test-retest reliability in both the waist-worn and midsole-worn pedometers during walking in our study.

The accuracy and reliability of the wrist-worn pedometer in counting steps improved from walking to running. The current study observed that the mean absolute precent error decreased from 9.09 to 2.98% and the intra-device reliability value increased from 0.82 to 0.95 during walking compared to running. These observations were consistence with previous works^33,34^. For instance, a study reported that the mean absolute precent error decreased from 6.30% during treadmill walking (5 min) to 2.58% during treadmill running (5 min), while reliability increased from 0.54 during treadmill walking to 0.85 during treadmill running^33^. This improvement could be attributed to increased locomotion speed, which results in higher peak angular momentum of arm movements, facilitating more accurate detection of wrist acceleration by the pedometer^35,36^. However, the midsole-worn pedometer still presented significantly higher accuracy in counting steps compared to the wrist-worn pedometer during running. The midsole-worn pedometer may have a movement pattern similar to the foot, allowing for clear changes in acceleration and facilitating more accurate detection of steps. Additionally, mean absolute percent error and Bland-Altman plots indicated that the waist-worn pedometer also presented higher accuracy in counting steps compared to the wrist-worn pedometer during running. When worn on the waist, pedometer is in close proximity to the body’s center of mass, aiding in the accurate detection of changes in acceleration during running^34^.

Only the midsole-worn pedometer presented acceptable reliability and significantly higher accuracy than the wrist-worn pedometer during stair climbing. Furthermore, according to the mean absolute percent error and Bland-Altman plots in the current study, the accuracy in counting steps was ranked as the midsole-worn > waist-worn > wrist-worn pedometers during stair climbing. Previous studies also reported poor accuracy for the wrist-worn and waist-worn pedometers in counting steps during stair climbing^25,26,37,38^. For instance, one study indicated that the wrist-worn pedometer (Fitbit zip) underestimated step count by 6.0 ± 10.0% during a total of 88 flights of stairs^26^. In addition, when worn on the waist, pedometer presented a mean absolute precent error of 11.4% and 9.4% during ascending & descending stairs, respectively^38^. Interestingly, the waist-worn pedometer presented an overestimated step count during stair climbing in the current study. The possible explanation is that stair climbing significantly influences lower limb movement characteristics due to greater forces on the knee and hip compared to overground walking^39^. Additionally, stair climbing produces greater changes in ankle joint kinematic compared to overground walking during the swing phase^27^. Hence, this kinematic feature in ankle joint may enhance the accuracy of the midsole-worn pedometer in counting steps during stair climbing.

In free-living conditions, the wrist-worn pedometer may record a higher number of steps compared to the waist-worn and midsole-worn pedometers^16,40^. These observations contradict our observations under laboratory conditions. This difference can be attributed to household activities that involve significant arm movements without corresponding stepping, which contribute to the step count only in wrist-worn pedometers^40^. Therefore, future research should consider evaluating the accuracy of midsole-worn pedometers in free-living conditions.

In conclusion, the current results exhibited higher accuracy and better reliability in the midsole-worn pedometer compared to the wrist-worn and waist-worn pedometers, especially during stair climbing. These observations suggest that the midsole-worn pedometers could offer comprehensive information about physical activities in laboratory environments. Additionally, the wrist-worn pedometer presented low accuracy and reliability in estimating steps during walking and stair climbing, which is not desirable as the appropriate body position to place pedometers in estimating steps.

## Methods

### Participants

The sample size was determined based on previous similar studies, which suggested a minimal sample size of 20^41,42^. The exclusion criteria included as follows: (1) body mass index beyond 32 kg/m^2^, (2) inability to running at least 400 m without an assistance, (3) any history of lower limb deficiency, such as limb or pelvis hypoplasia or aplasia syndrome, and (4) any history of musculoskeletal injuries or surgery at the lower limb in the last six months. Snowball sampling strategy was used to recruit participants. All methods were conducted in accordance with the Declaration of Helsinki. This study was approved by the Ethics Committee of an East Asian University (NO. 102772023RT099). All eligible participants signed a written informed consent form prior to testing.

### Instruments

Three commercial pedometers were used in this study. Specifically, one Fitbit Zip pedometer (Fitbit Company, San Francisco, USA) was placed on participants’ posterior superficial right wrist, located in the middle of the ulnar and radial styloids (wrist-worn). Another Fitbit Zip pedometer was attached to participants’ sacrum, located in the middle of posterior superior iliac spine (waist-worn). These two pedometers were fixed using the flexible bandage by one investigator throughout the study. The Fitbit Zip has been demonstrated to be a valid measure of step counts in free-living physical activity in healthy adults^43^. The third commercial pedometer (Xunying, 361°, Xiamen, China) was embedded into the shoe’s midsole at the right heel (midsole-worn). All these pedometers were accelerometer-based.

### Procedures

Participation in this study required two visits on separate days. After the first visit, participants were scheduled for a second visit 7 days later. During each visit, three different conditions were performed by our participants: (1) walking, (2) running, and (3) stair climbing. Each condition was performed once. For the walking and running conditions, participants walked and ran around a 400-m outdoor track. For the stair climbing condition, participants continuously ascended and descended 17-step staircase, repeating this ten times. Participants used their preferred speed for each condition.

Each condition started with the participants standing quietly upon hearing “ready” from the investigator. Then, the instruction “go” was given for participants to begin the condition, followed by the instruction “stop” at the end of each condition. Participants were instructed to stand as still as possible to record the number of steps from the pedometers when hearing “ready” and “stop”. Meanwhile, the number of steps was simultaneously recorded by a video camera (gold standard) during the first visit to evaluate the accuracy of each pedometer in different locations of attachment. During the second visit, the number of steps was recorded only by the pedometers to evaluate the reliability of each pedometer in different locations of attachment. Instructions for the second visit were the same as for the first visit.

### Data analysis

The number of steps recorded by the pedometers from different locations of attachment was calculated using the formula: recorded number at the ending point – recorded number at the onset point. The actual steps were manually counted by two investigators according to the video as the gold standard. The playback speed of the video was set to ½ speed. The criterion was set to have the same number of steps from the two investigators. If the number of steps counted by the two investigators was different, the same procedure was performed again 7 days later until the same results were obtained. The difference between the number of steps from the pedometers and the number of steps recorded by the video (|steps from the pedometers - gold standard|) was calculated as the error scores.

### Statistical analysis

The baseline characteristics of the three pedometers at different locations of attachment and gold standard were summarized using the median and interquartile range (IQR). To determine the accuracy of each pedometer in different locations of attachment, three one-way ANOVAs were used to investigate the wearing location difference in error scores during walking, running, and stair climbing. If applied, post hoc analysis with Bonferroni adjustments was used for multiple comparisons among the three different locations of attachment. Then, Cohen’s d effect size was calculated to interpret the size of the difference between two locations of attachment. A significant level was set at 0.05.

Additionally, the mean percent error (MPE) was also calculated to determine the pedometers’ accuracy by using the following formula: ∑((steps from the pedometers –gold standard)/gold standard × 100)/number of participants. A positive result reflects a possibility in overestimated number of steps, whereas a negative result reflects a possibility in underestimated number of steps^44^. The mean absolute percent error (MAPE) is further calculated from the average of the absolute percent errors. The higher MAPE value reflects a less accurate in estimating number of steps. Bland-Altman plots were plotted allow visual evaluation of bias or trends between measured steps from the pedometers and the gold standard^45^. The closer the mean difference is to zero, the better the agreement between measured steps from the pedometers and the gold standard^46^.

To identify the reliability of each pedometer in different locations of attachment between first and second visits, Cronbach’s alpha value was calculated to assess intra-device reliability during walking, running, and stair climbing. The Cronbach’s alpha values in the ranges of 0.70–0.90 and 0.91 or more were considered to represent acceptable and strong reliability, respectively^47^. All statistical analyses were conducted using the SPSS system (19.0, SPSS Inc., Chicago, IL, USA).

## Electronic supplementary material

Below is the link to the electronic supplementary material.

Supplementary Material 1

## Data Availability

All data generated or analyzed during this study are included in this published article’s supplementary material.
